# A novel nomogram based on cell cycle-related genes for predicting overall survival in early-onset colorectal cancer

**DOI:** 10.1186/s12885-023-11075-y

**Published:** 2023-06-27

**Authors:** Meijuan Xiang, Yuan Gao, Yue Zhou, Muqing Wang, Xueqing Yao

**Affiliations:** 1 School of Medicine, South China University of Technology, Guangzhou, 510006 China; 2 Department of Gastrointestinal Surgery, Guangdong Provincial People’s Hospital, Guangdong Academy of Medical Sciences, Guangzhou, 510080 China; 3 Department of General Surgery, Guangdong Provincial People’s Hospital Ganzhou Hospital (Ganzhou Municipal Hospital), Ganzhou, 341000 China; 4 Department of General Surgery, Foresea Life Insurance Shaoguan Hospital, Shaoguan, 512000 China; 5 The Second School of Clinical Medicine, Southern Medical University, Guangzhou, 510515 China

**Keywords:** Young-onset colorectal cancer, Cell cycle, Prognostic indicator, Individualized treatment

## Abstract

**Background:**

Although the incidence of late-onset colorectal cancer (LOCRC) has decreased, the incidence of early-onset colorectal cancer (EOCRC) is still rising dramatically. Heterogeneity in the genomic, biological, and clinicopathological characteristics between EOCRC and LOCRC has been revealed. Therefore, the previous prognostic models based on the total CRC patient population might not be suitable for EOCRC patients. Here, we constructed a prognostic classifier to enhance the precision of individualized treatment and management of EOCRC patients.

**Methods:**

EOCRC expression data were downloaded from the Gene Expression Omnibus (GEO) and The Cancer Genome Atlas (TCGA) databases. The regulatory pathways were explored by gene set enrichment analysis (GSEA). The prognostic model was developed by univariate Cox-LASSO-multivariate Cox regression analyses of GEO samples. TCGA samples were used to verify the model. The expression and mutation profiles and immune landscape of the high-risk and low-risk cohorts were analyzed and compared. Finally, the expression and prognostic value of the model genes were verified by immunohistochemistry and qRT‒PCR analysis.

**Results:**

The cell cycle was identified as the most significantly enriched oncological signature of EOCRC. Then, a 4-gene prognostic signature comprising *MCM2, INHBA, CGREF1*, and *KLF9* was constructed. The risk score was an independent predictor of overall survival. The area under the curve values of the classifier for 1-, 3-, and 5-year survival were 0.856, 0.893, and 0.826, respectively, in the training set and 0.749, 0.858, and 0.865, respectively, in the validation set. Impaired DNA damage repair capability (*p* < 0.05) and frequent *PIK3CA* mutations (*p* < 0.05) were found in the high-risk cohort. CD8 T cells (*p* < 0.05), activated memory CD4 T cells (*p* < 0.01), and activated dendritic cells (*p* < 0.05) were clustered in the low-risk group. Finally, we verified the expression of MCM2, INHBA, CGREF1, and KLF9. Their prognostic value was closely related to age.

**Conclusion:**

In this study, a robust prognostic classifier for EOCRC was established and validated. The findings may provide a reference for individualized treatment and medical decision-making for patients with EOCRC.

**Supplementary Information:**

The online version contains supplementary material available at 10.1186/s12885-023-11075-y.

## Introduction

Colorectal cancer (CRC) is a very common malignant tumor worldwide [1]. The overall incidence and mortality rates of CRC have decreased globally. However, the morbidity of early-onset colorectal cancer (EOCRC) has increased substantially. EOCRC is usually defined as colorectal cancer diagnosed in patients under 50 years old [2–5]. It is projected that EOCRC will account for approximately 10 to 12% of colon cancer and 25% of rectal cancer diagnoses by 2030 [4, 6], which highlights that EOCRC represents a large cancer burden among young people [2]. Compared with late-onset CRC (LOCRC), which is diagnosed in patients ≥ 50 years old, EOCRC is characterized by strong aggressiveness, high malignancy, and late disease staging in the clinic [7–9]. In terms of molecular and genetic features, the VEGF, EGF, and WNT pathways are overexpressed in MSS-EOCRC, while low expression is observed in LOCRC, but expression patterns vary with age [10]. High hypomethylation is a unique feature of EOCRC (*P* < 0.0001), reflecting whole genome hypomethylation and chromosomal instability [11, 12]. MSI in EOCRC patients is usually related to MSH2 inactivation, while in LOCRC patients, it is usually related to MLH1 inactivation [13]. Oxidation‒reduction imbalance was found to be a distinct molecular feature of EOCRC patients in Europe [14]. Yanlei Ma et al. [15] identified distinct microbiome–metabolome associations in LOCRC and EOCRC. Sherman SK et al. [16] constructed a biological bank containing 20 EOCRC organoids, detected key gene mutations and transcriptome changes, observed significant molecular phenotypic diversity, including PTPRK-RSPO3 fusion, and revealed that EOCRC has different genetic profiles and distinct synergistic pathways. Therefore, EOCRC should be evaluated, managed, and investigated separate from LOCRC [3, 4, 17].

The cell cycle refers to the entire process that a continuously dividing cell undergoes from the end of one mitosis process to the end of the next. Maintaining the integrity of the genome is crucial for chromosome separation and cell proliferation [18]. The cell cycle is a process strictly regulated by multiple control mechanisms to ensure the creation of two euploid cells with the same gene [19]. Errors in the separation mechanism during cell division lead to the existence of abnormal chromosomes, resulting in aneuploid cells, which are related to many cancer cells and lead to genome imbalance [20, 21]. Aneuploidy is considered one of the driving mechanisms of tumorigenesis [22]. The continuous proliferation signal leads to excessive cell division, which is the hallmark of cancer [23]. Mutations in signaling pathways that initiate exit from the cell cycle or promote S phase entry result in sustained cell division in cancer cells [24]. The key regulator of cell cycle processes is the activity of cyclin-dependent kinases (CDKs) [23]. In the early G1 phase, CDKs determine whether a cell remains in the cell cycle or exits [23]. Increased CDK activity has been widely reported in various cancers [25]. CDK inhibitors may force cancer cells to permanently exit the cell cycle, thus preventing sustained cell proliferation and inhibiting tumor growth [26]. In summary, abnormalities in the core mechanism of the cell cycle exist in almost all tumor types and represent a driving force of tumorigenesis [27].

The prognosis of EOCRC is still controversial. Patients with EOCRC often receive more radical treatment, but unfortunately, not all patients experience a benefit [28–30]. Thus, further study on the prognostic indicators of EOCRC is of great significance for individualized management. Due to the distinct genetic and molecular features between EOCRC and LOCRC [11–16], a previously established prognostic signature based on all CRC data [31, 32] might not yield an accurate prediction for EOCRC patients. To the best of our knowledge, few studies have focused on the potential factors that affect the survival of EOCRC at the molecular level.

Thus, we processed and analyzed the transcriptome data of early-onset colorectal cancer from the GEO and TCGA databases and combined these data with clinical and pathological factors to build a precise classifier to predict the overall survival (OS) of EOCRC patients. This was followed by experimental verification. In conclusion, our study may provide novel insights regarding clinical prognosis assessment and potential individualized treatment for patients with EOCRC.

## Materials and methods

### Patient selection

CRC patients in the Department of Gastrointestinal Surgery, Guangdong Provincial People's Hospital from January 2017 to March 2018 were enrolled to analyze the clinical features of EOCRC. Patients with solitary primary colorectal carcinoma without a history of cancer who did not receive any preoperative neoadjuvant therapy followed by radical resection and who had complete clinical and survival data were our target population. Information on sex, age, TNM stage, and tumor location was obtained from the patient's medical records. This study complied with the Helsinki Declaration and was approved by the author's organization. All experiments followed relevant regulations. All enrolled CRC patients provided written informed consent, and the collection of the clinical sample was approved by the Institutional Review Board of Guangdong Provincial People's Hospital under grant number GDREC2019504H (R2).

### Data collecting and preprocessing

The gene microarray and clinical data of EOCRC were downloaded from the Gene Expression Omnibus (GEO) (https://www.ncbi.nlm.nih.gov/geo/). Moreover, the RNA-Seq and corresponding clinical information were downloaded from the TCGA website (https://portal.gdc.cancer.gov/).

GEO Series (GSE) 41,258 (31 EOCRC samples versus 10 control samples), GSE87211 (19 EOCRC samples versus 14 control samples), and TCGA (69 EOCRC samples versus 51 control samples) data were used to find the differentially expressed genes between EOCRC and normal cohorts. Patients had to meet the following criteria: (1) both the age of patients whose tumor and nontumor samples were used should be < 50 years for the GEO; and (2) tumor samples should be from patients < 50 years at diagnosis and all normal samples should be from patients < 50 years for the TCGA.

Moreover, data for 202 EOCRC patients from GSE41258 (31 patients), GSE39582 (66 patients), GSE17536 (20 patients), GSE17537 (9 patients), GSE12945 (7 patients), and TCGA (69 patients) datasets, were obtained for survival research; the included patients met the following criteria: (1) tumor patients who were diagnosed when they were < 50 years of age; (2) patients with available survival information; and (3) patients with survival time > 30 days. The clinical information of samples from the GEO and TCGA datasets is presented in Supplementary Table 1.

In addition, mutation data for 63 patients were obtained from the TCGA database.

### Identification of the most significant differentially expressed genes in EOCRC

GSEA was applied to investigate the clusters of significant differentially expressed genes between EOCRC and normal tissues in GSE41258. Pathways with a false nominal *p* value < 0.05, discovery rate (FDR) q-value < 0.25, and normalized enrichment score (NES) > 1 were regarded as significantly enriched.

All cell cycle-related genes were screened from the molecular signatures database (MSigDB) (http://software.broadinstitute.org/gsea/msigdb). Then, a novel complete cell cycle gene set consisting of 2579 unique genes was generated after assembling and removing duplications.

Finally, differentially expressed genes (DEGs) between EOCRC and normal samples in the GSE41258, GSE87211, and TCGA datasets were identified using the “DESeq2” package in R [33]. |Log2FC|> 1 and an FDR < 0.05 were set as the thresholds. Then, the common DEGs were identified by integrating the respective DEGs of the three datasets. Finally, the EOCRC cell cycle-related DEGs were identified by integrating the cell cycle genes and the common DEGs.

### Removal of the batch effect

Since the data are derived from different cohorts, the RNA expression data of GSE41258, GSE39582, GSE17536, GSE17537, GSE12945, and TCGA were log10-transformed and normalized using the “limma” R package [34] and were corrected for the batch effect by the “sva” package in R [35].

### Establishment of a cell cycle prognostic classifier

GEO samples were used as the training group on account of their larger sample size. First, survival-related cell cycle genes were found by univariate Cox analysis (*p* < 0.05). Then, to prevent model overfitting, the least absolute shrinkage and selection operator (LASSO) algorithm [36] was employed to remove highly correlated genes. Ultimately, a four-gene prognostic signature was established through the Cox proportional hazards model method.

According to the median risk score calculated by the signature, samples were divided into high-risk and low-risk groups. We compared the prognosis between the high- and low-risk cohorts with the packages "survival" and "survminer", and a significant p value was obtained. Kaplan–Meier survival curves and receiver operating characteristic (ROC) curves for OS were plotted to test the applicability of the classifier. The independent clinicopathological factors affecting OS were found by multivariate Cox analysis (*p* < 0.05). Next, a nomogram was constructed. Finally, the calibration curve was utilized to assess the nomogram. As an external validation group, the TCGA samples were assigned to the high-risk group and low-risk group on the basis of the same median risk score of the training group. The Kaplan–Meier curve, ROC curve, and calibration curve were also plotted to validate the model.

### Pathway enrichment analysis

Gene set variation analysis (GSVA) [37] could identify the different upregulated gene clusters for each sample, in which a variety of pathway activities over a sample population are applied in an unsupervised manner. KEGG [38–40](c2.cp.kegg.v7.4.symbols.gmt), GO (c5.go.v7.4.symbols.gmt), and HALLMARK (h.all.v7.4.symbols.gmt) were chosen as the reference files. Significantly enriched pathways between the different EOCRC risk cohorts were screened out by the “GSVA” and "limma" packages. |log2FC|> 0.15 and FDR < 0.05 were the criteria for a significant differentially enriched pathway.

### Mutation and immune cell infiltration analysis

The "maftools" package [41] was applied to explore the mutation differences between the high-risk and low-risk cohorts. Since only 63 EOCRC samples from the TCGA dataset had mutation data, mutation analysis between high-risk and low-risk groups was conducted only on these 63 EOCRC samples. CIBERSORT [42] was used to evaluate the abundances of tumor-infiltrating immunocytes between the high-risk and low-risk groups of the 202 early-onset CRC samples because this algorithm could robustly distinguish twenty-two types of human immunocytes based on genetic expression data of miscellaneous cells.

### Validation of the genes via public data analysis tools

GEPIA (http://gepia.cancer-pku.cn) was used to validate the expression levels of the model genes between colorectal carcinoma and normal tissues. GEPIA data come from the TCGA and GTEx databases, including 275 colon cancer samples and 349 normal colon samples, 92 rectal cancer samples and 318 normal rectal samples.

### Immunohistochemistry analysis

A total of 81 colorectal adenocarcinoma surgical samples were collected from patients who underwent radical surgery at Guangdong Provincial People's Hospital from January 2016 to March 2018. The inclusion criteria were as follows: (1) qualified tissue specimen quality; (2) complete follow-up data; (3) sporadic CRC. The exclusion criteria were as follows: (1) missing or poor-quality tissue specimens (2) incomplete follow-up data. The Institutional Review Committee of Guangdong Provincial People's Hospital approved the collection of tissue samples and clinical data. All included patients signed informed consent documents.

The cancerous tissue was dewaxed in xylene solution for 15 min. Then, a series of graded ethanol solutions (100%, 95%, 80%, and 70%) were applied for 5 min each. Then, antigen repair was carried out at a high temperature and high pressure for 5 min. Peroxidase inhibitor was applied at 37 °C for 20 min. The primary antibody was incubated at 4 °C overnight. The secondary antibody was added and incubated at room temperature for 1 h. DAB chromogenic solution was developed under an electron microscope, hematoxylin was stained for 5 min, and ethanol hydrochloride was differentiated for 1 min. Resin and cover glass were used to seal the film and photographed under an electron microscope. The staining scores were as follows:—for negative, + for weakly positive, +  + for moderately positive, and +  + for strongly positive. Staining scores as—or + was considered to represent low expression, and staining scored as +  + or +  +  + was considered to represent high expression. Antibodies and dilutions were as follows: INH3A (1:250, D220861, Sangon Biotech, Shanghai China), CGREF1 (1:250, D124529, Sangon Biotech, Shanghai, China), KLF9 (1:500, ab227920, Abcam, MA, USA), and MCM2 (1:250, D120962-0025, Sangon Biotech, Shanghai, China).

### Quantitative Real-Time PCR analysis

Six EOCRC and six LOCRC samples were randomly selected from samples obtained from CRC patients who underwent radical surgery at Guangdong Provincial People's Hospital from January 2017 to March 2018. All enrolled patients underwent radical surgery, and the postoperative pathology was adenocarcinoma. The Institutional Review Committee of Guangdong Provincial People's Hospital approved the collection of tissue samples and clinical data. All included patients signed informed consent documents.

Total RNA was extracted using the RaPure Total RNA Kit (Magen Biotechnology Co., Ltd), and cDNA synthesis was performed using the HiFiScript cDNA Synthesis Kit (CoWin Biosciences, Ltd), after which Hieff® qPCR SYBR Green Master Mix (No Rox) was used for qPCR (Yeasen Biotechnology (Shanghai) Co., Ltd.). The relative identified mRNA expression levels were normalized to glyceraldehyde 3-phosphate dehydrogenase (GAPDH) and calculated with the 2–ΔΔCT method. The BLAST tool was applied to verify the specificity of the designed primers, the sequences of which are shown in Supplementary Table 4. All experiments were performed in triplicate.

### Statistical analysis

Statistical analyses were conducted using R version 4.0.2 (https://www.r-project.org/) and GraphPad Prism version 8.4.3 (Dotmatics, San Diego, CA, USA). Categorical variables were analyzed with the chi-square or Wilcoxon rank-sum test. Continuous variables were analyzed with Student's t test. Kaplan‒Meier analysis estimated the cumulative OS. The log-rank test was used to compare the survival curves. OS referred to the time from tumor resection to patient death. Factors associated with OS were calculated by the Cox proportional hazards model and indicated by hazard ratio (HR) and 95% confidence interval (CI). The time-dependent receiver operating characteristic curve was constructed and the area under the curve (AUC) was determined by the "timeROC" R package. The calibration curve was plotted by the "rms" package. Mutations in the high- and low-risk groups were assessed by the Fisher test in the "maftools" R package. The p values of for DEG analysis, GSEA, and GSVA were adjusted based on multiple testing corrections. Unless otherwise stipulated, *p* < 0.05 was considered to indicate statistical significance.

## Results

### Clinical characteristics of the EOCRC cohort

In all, 273 CRC patients (EOCRC = 42 samples and LOCRC = 231 samples) who accepted curative surgery from January 2017 to March 2018 at our hospital were included in the analysis of the clinical characteristics (Table 1). We found that EOCRC patients had a later TNM stage and worse prognosis than LOCRC patients (*p* < 0.05).

**Table 1:** The clinicopathological features of the enrolled colorectal cancer patients who underwent radical surgery at Guangdong Provincial People's Hospital from January 2017 to March 2018

Clinical features	EOCRC	LOCRC	*p*-value
**Age**	< 50	≥ 50	
**Sex**			
Female	22	93	
Male	20	138	0.143
**T**			
1	3	17	
2	13	59	
3	23	141	
4	3	14	0.663
**N**			
N0	15	127	
N1	18	73	
N2	9	14	0.001
**M**			
M0	36	208	
M1	6	23	0.403
**Stage**			
I ~ II	15	127	
III ~ IV	27	104	0.022
**Location**			
Left colon	15	69	
Right colon	8	46	
Rectum	19	116	0.746
**Vital status**			
Alive	36	208	
Dead	6	23	0.022

Abbreviations: *LOCRC* late-onset colorectal cancer, *EOCRC* early-onset colorectal cancer

### Identification of cell cycle DEGs

**Fig. 1:**
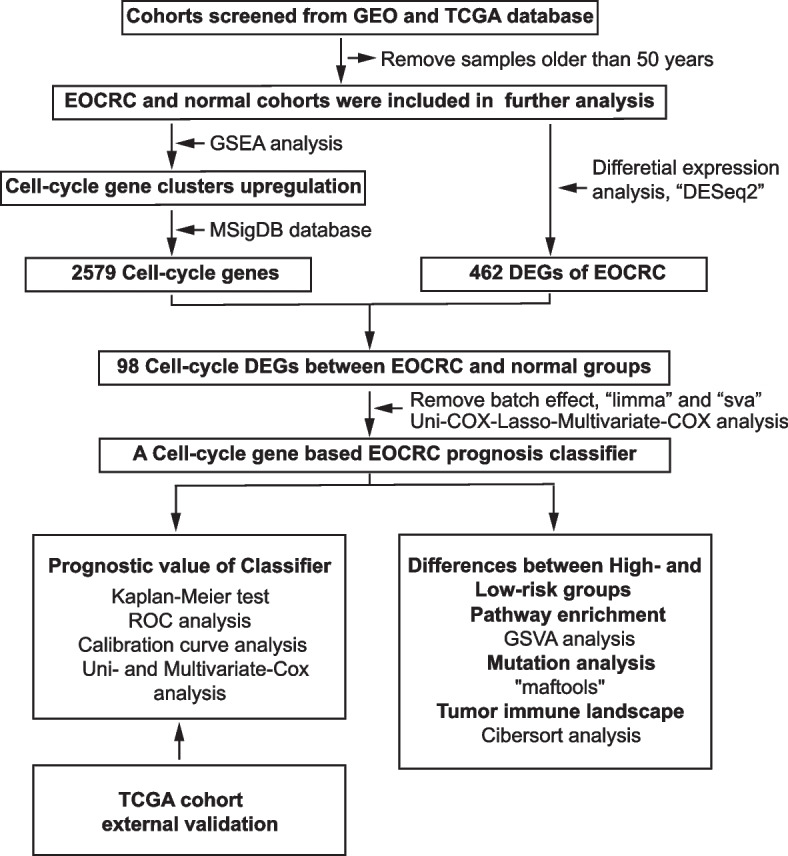
Flowchart presenting the process of bioinformatics analysis. Abbreviations: EOCRC, early-onset colorectal cancer; GSEA, gene set variation analysis and gene set enrichment analysis; GSVA, gene set variation analysis; DEGs, differentially expressed genes; ROC, receiver operating characteristic

**Fig. 2:**
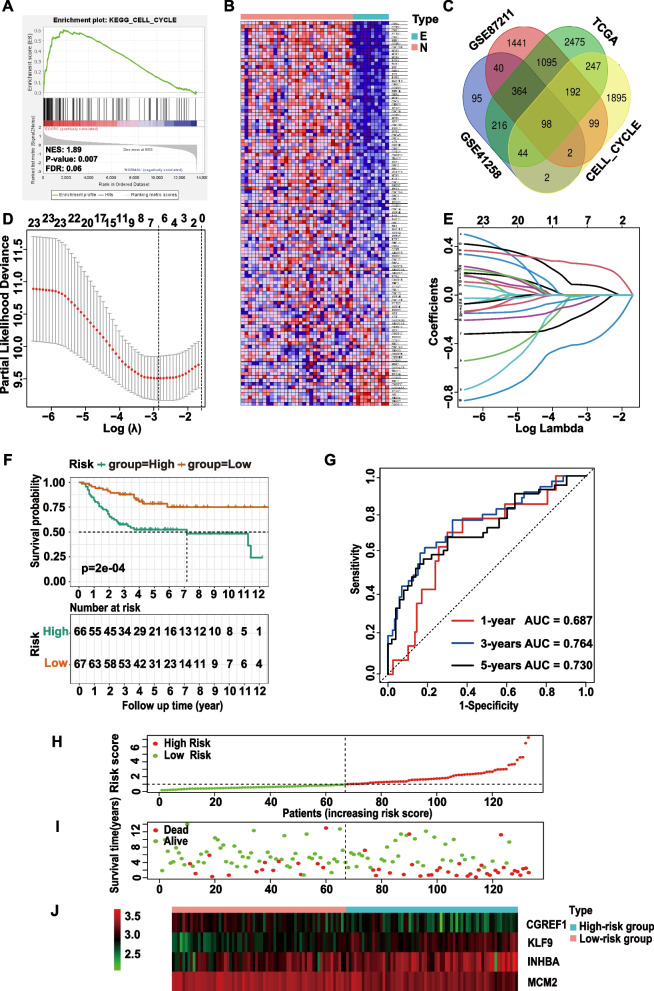
Construction of a cell cycle prognostic model for EOCRC. **A** The cell cycle pathway was identified as the most significantly enriched oncological signature of EOCRC in GSE41258 by GSEA. **B** Heatmap of cell cycle DEGs between the tumor and normal groups of GSE41258. **C** A Venn diagram indicates that 98 common cell cycle prognostic DEGs were identified in the GSE41258, GSE87211, and TCGA cohorts. **D** The LASSO Cox regression model was constructed from the 23 prognostic genes, and the tuning parameter (λ) was calculated based on the partial likelihood deviance with tenfold cross-validation. **E** The best log lambda value (corresponding to the minimum cross-validation error point) was selected for the training group in the LASSO model. **F** Kaplan–Meier survival analysis of the low- and high-risk group patients in the training cohort. **G** AUC value according to the 1-, 3-, and 5-year survival of the area under the ROC curve. **H**,** I** The distribution of risk scores and survival status in the training cohort are plotted and marked as low risk (green) or high risk (red). **J** The expression of the 4 model genes of each EOCRC patient in the training cohort by z score, with red indicating higher expression and light green indicating lower expression. Abbreviations: NES, normalized enrichment score; FDR, false discovery rate; E, early-onset colorectal cancer; N, normal; AUC, area under the curve; LASSO, least absolute shrinkage and selection operator; EOCRC, early-onset colorectal cancer; GSEA, gene set enrichment analysis; DEGs, differentially expressed genes; AUC, area under the curve; ROC, receiver operating characteristic

The flow chart of the bioinformatics analysis is shown in Fig. 1. Using KEGG as the background gene set for GSEA, it was found that compared with the normal cohort, the top three pathways with the highest NES in the EOCRC cohort were cell cycle, DNA replication, and base excision repair. When using hallmark genes as the background gene set, the top three gene sets with the highest NES in EOCRC were MYC TARGETS V1, G2M CHECKPOINT, and MYC TARGETS V2. Based on GO gene sets, the top three biological pathways most enriched in EOCRC were signal transduction in response to DNA damage, regulation of signal transduction by p53 class mediator, and regulation of ubiquitin protein ligase activity. In summary, these results showed that many cell cycle-associated gene sets were significantly upregulated in the EOCRC cohort of GSE41258 compared to the normal cohort (Figs. 2A, B, Supplementary Fig. 1A). Therefore, we believed that abnormal activity of cell cycle-related pathways is the most significant tumor feature of EOCRC. Therefore, we selected cell cycle genes as our research object. In all, 861, 3331 and 4731 DEGs were screened out between the EOCRC and normal groups from the GSE41258, GSE87211, and TCGA datasets, respectively. After integrating the above three groups of DEGs and 2579 cell cycle-related genes, 98 common cell cycle-related DEGs (76 upregulated and 22 downregulated genes) were identified for model construction (Fig. 2C, Supplementary Table 2).

### Construction and validation of the cell cycle prognostic nomogram

We removed the batch effect of the GSE41258, GSE39582, GSE17536, GSE17537, GSE12945, and TCGA datasets (Supplementary Figs. 1B, C) and filtered out 23 prognostic genes by univariate Cox regression analysis (*p* < 0.05) (Supplementary Table 3). After implementation of the LASSO algorithm with one standard error (SE) and 100-fold cross-validation (Figs. 2D, E), 6 significant prognostic genes (*KLF9, INHBA, MCM2, CGREF1, MLXIPL, TUBAL3*) were found. Following stepwise multivariate Cox proportional hazards regression analysis, a 4-cell cycle gene prognostic signature was established.

$$risk\;score=\;KLF9\ast(0.325369249701883)+\;INHBA\ast(0.15674709153344)+CGREF1\ast(-0.285912665465943)+MCM2\ast(-\;0.547574857455513)$$

According to the median risk score, the GEO training cohort was classified into high- and low-risk groups. The Kaplan–Meier survival curve suggested that patients in the high-risk group had poor OS (Fig. 2F). The AUCs for 1-, 3-, and 5-year survival of the signature were 0.687, 0.764, and 0.730, respectively (Fig. 2G). The C-index was 0.807. As shown in Figs. 2H-J, the differences in the expression of model genes and patient survival were obvious between the high- and low-risk EOCRC groups.

**Fig. 3:**
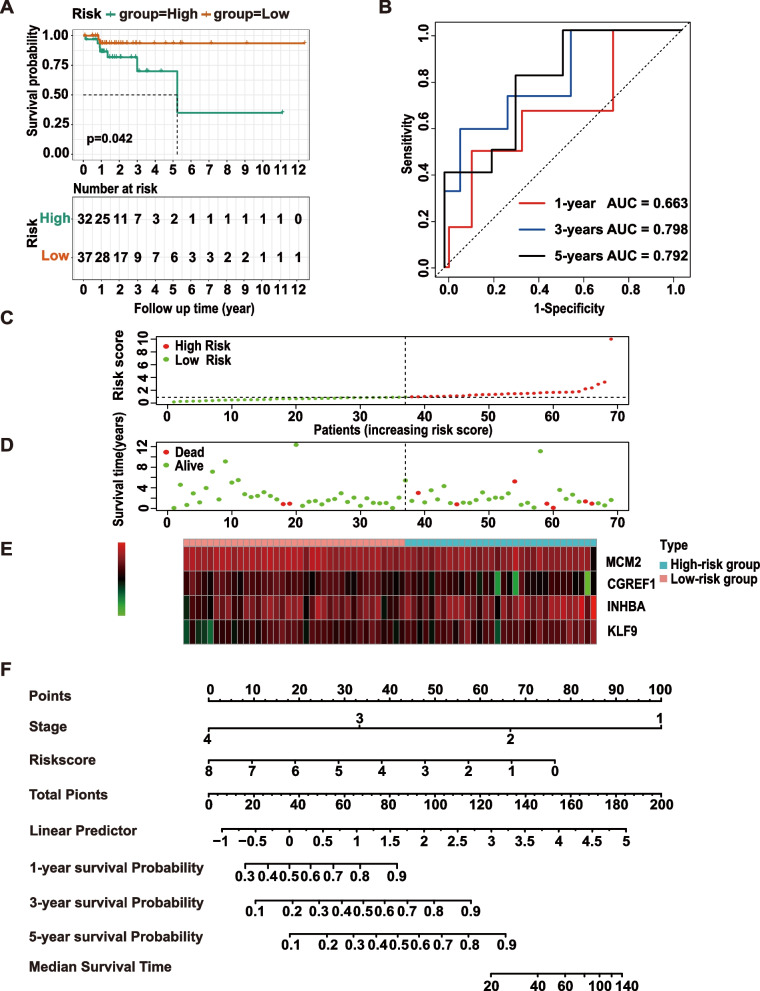
Validation of the signature and construction of a nomogram. **A** Kaplan–Meier survival analysis of the low- and high-risk group patients in the TCGA external validation cohort. **B** AUC values for 1-, 3-, and 5-year survival in the TCGA cohort. **C**,** D** The distribution of risk score and survival status in TCGA are plotted and marked as low risk (green) or high risk (red). **E** The expression of the 4 model genes in the validation cohort between the high- and low-risk groups. **F** A nomogram for predicting the OS of EOCRC patients based on the risk score and tumor stage was confirmed. Abbreviations: AUC, area under the curve; ROC, receiver operating characteristic; OS, overall survival; EOCRC, early-onset colorectal cancer

To validate the OS predictive value of the classifier, the TCGA EOCRC cohort (*n* = 69) was used as the external validation set and had the same risk formula and cutoff point as the GEO cohort. The high-risk group had markedly poorer outcomes (Fig. 3A), which was in agreement with the results for the training set. The AUCs for 1-, 3-, and 5-year survival were 0.663, 0.798, and 0.792, respectively. (Fig. 3B). The risk score could also distinguish high- and low-risk groups excellently in the TCGA cohort, with significant differences in prognosis and model gene expression levels between the two groups (Fig. 3C-E). The risk score (HR 1.279, 95% CI 1.075–1.522, *p* = 0.005) and tumor stage (HR 3.484, 95% CI 2.184–5.557, *p* < 0.001) were confirmed as independent predictors by univariate and multivariate Cox regression analyses. Then, a nomogram based on the risk score and tumor stage was constructed and assessed (Fig. 3F).

**Fig. 4:**
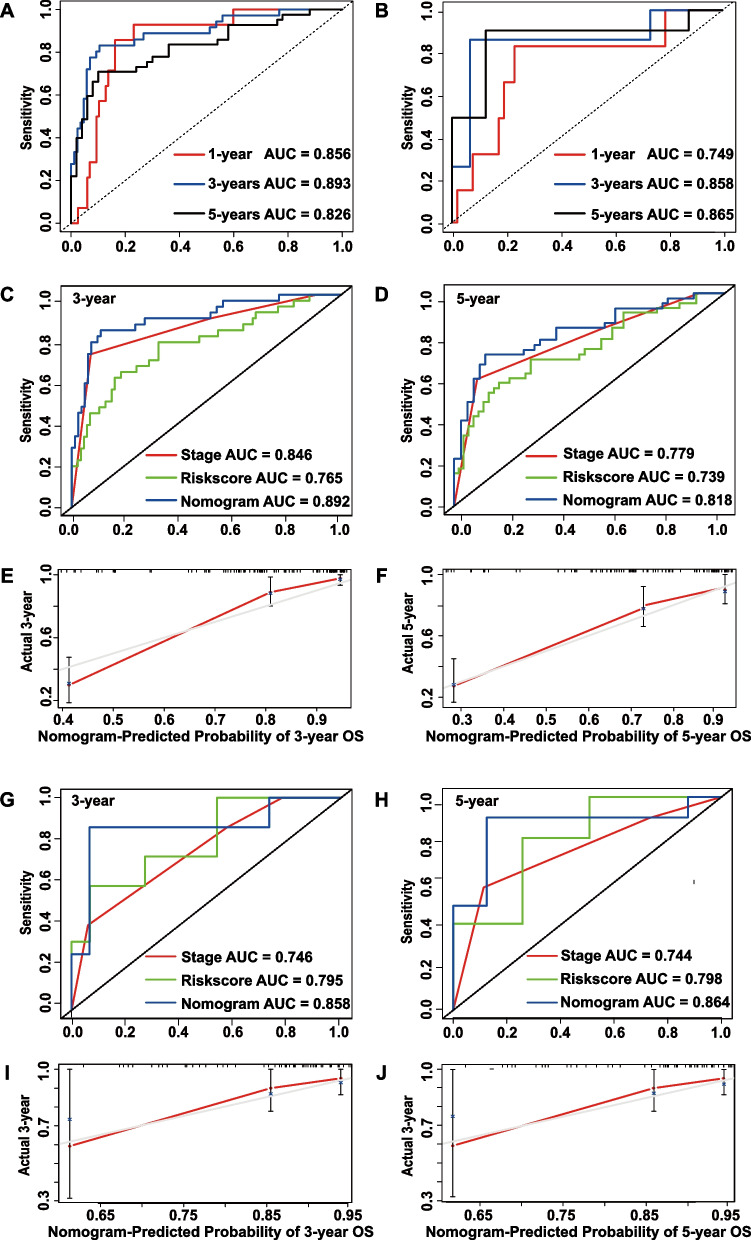
The prediction efficiency of the model in the training and validation sets. **A**,** B **Time-dependent ROC curves and AUC values showed the predictive ability of the nomogram in terms of 1-, 3-, and 5-year survival in the training set **(A)** and validation set **(B)**. **C**,** D** Time-dependent ROC curve analyses demonstrated that the nomogram performed better than tumor stage and the 4-gene signature alone in the GEO cohort at 3 and 5 years; the same findings was observed for the TCGA cohort **G**,** H**. The calibration plots for predicting 3- and 5-year OS of the GEO cohort **E**,** F** and TCGA cohort **I**,** J**. Abbreviations: AUC, area under the curve; ROC, receiver operating characteristic; OS, overall survival

The AUCs of the nomogram of the GEO set reached 0.856, 0.893, and 0.826 at 1, 3, and 5 years, respectively (Fig. 4A). The AUCs of the TCGA set were similar at 0.749, 0.858, and 0.865 (Fig. 4B). The nomogram showed better predictive performance than the signature or tumor stage alone in both the GEO cohort (Figs. 4C, D) and the TCGA cohort (Figs. 4G, H). The calibration curve was used to assess the precision and sensitivity of the prognostic nomogram for EOCRC patients in both the training (Figs. 4E-F) and validation groups (Figs. 4I-J).

### Comparison of clinicopathological and molecular characteristics between the high-risk and low-risk groups

The clinicopathological features between the identified high-risk and low-risk cohorts were compared, and patients in the high-risk group in the GEO cohort had advanced TNM stage (stage III/IV, *p* < 0.01), while patients in the TCGA cohort showed no significant difference in TNM stage (*p* = 0.298).

**Fig. 5:**
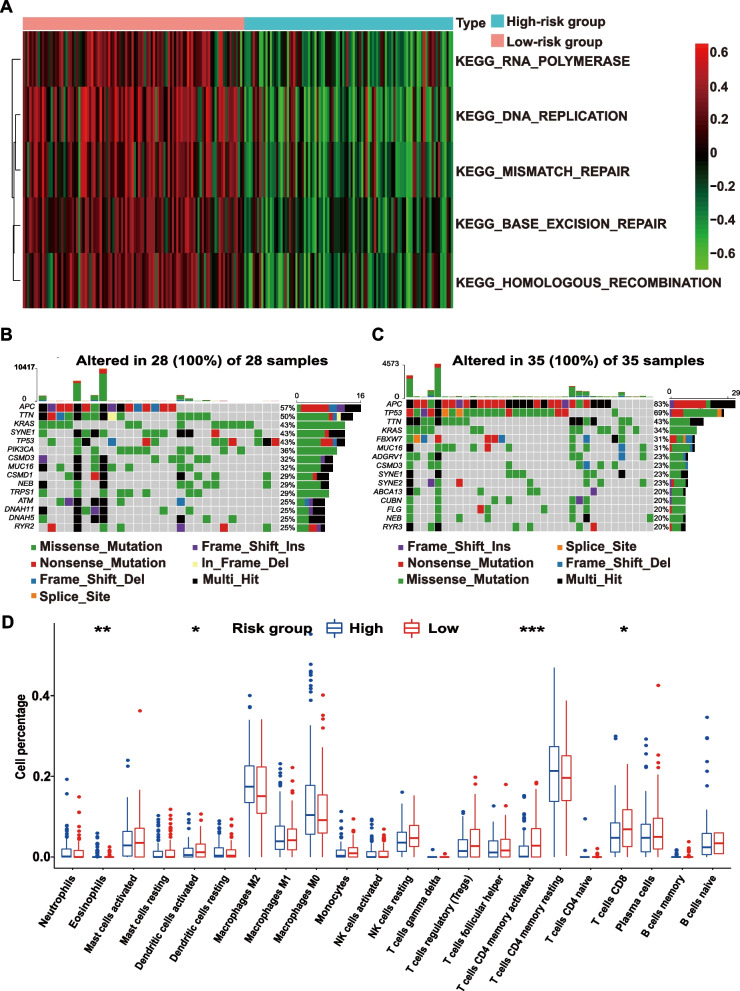
Differences in the biological mechanism, mutation, and TIL profiles between the high- and low-risk groups. **A** GSVA identified the top 5 downregulated pathways in the high-risk group compared with the low-risk group based on KEGG. **B**,** C** Mutation difference between high- (B) and low-risk **C** cohorts in TCGA. **D** Comparison between the fractions of immune cells in the high- and low-risk groups of the entire EOCRC cohort via the CIBERSORT method. *p < 0.05;**p < 0.01; ***p < 0.001; ****p < 0.0001. Abbreviations: TIL, tumor-infiltrating lymphocyte; GSVA, gene set variation analysis; EOCRC, early-onset colorectal cancer

The GSVA based on KEGG gene sets showed that RNA polymerase, DNA replication, mismatch repair, base excision repair, and homologous recombination were obviously downregulated in the high-risk EOCRC cohort (Fig. 5A). It also confirmed by GO analysis of the GSVA results that signaling pathways that enhance anticancer activity were significantly enriched in the low-risk cohort (Supplementary Fig. 1D).

As shown in the mutation waterfall plot (Figs. 5B, C), the top three most commonly mutated genes in the high-risk group were *APC* (57%), *TTN* (50%), and *KRAS* (43%), while those in the low-risk group were *APC* (83%), *TP53* (69%) and *TTN* (43%). *PIK3CA* mutations were more frequent in the high-risk group than in the low-risk group (36% vs. 14%, *p* < 0.05).

CIBERSORT revealed the immune landscape of the two groups. As shown in Fig. 5D, CD8 T cells (*p* < 0.05), activated memory CD4 T cells (*p* < 0.01) and activated dendritic cells (*p* < 0.05) were significantly enriched in the low-risk cohort, while eosinophils (*p* < 0.05) were enriched in the high-risk group.

### MCM2, KLF9, INHBA, and CGREF1 were significant prognostic indicators for EOCRC patients

**Fig. 6:**
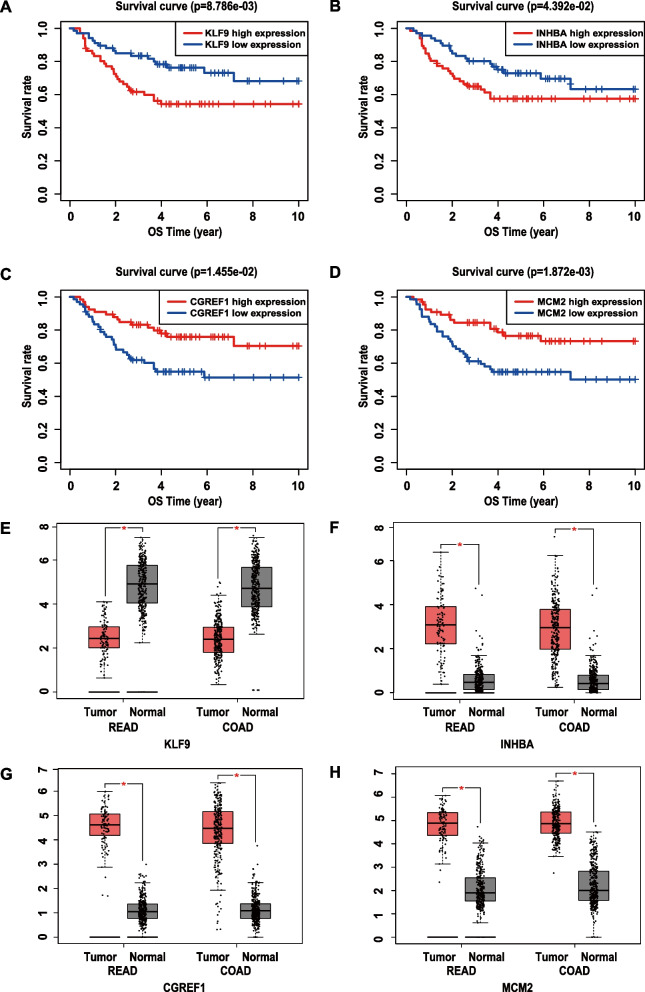
The expression of MCM2, KLF9, INHBA, and CGREF1 and their prognostic significance. **A-D** Overall survival analysis is based on the expression levels of MCM2, KLF9, INHBA, and CGREF1 in EOCRC. **E–H** The expression of MCM2, KLF9, INHBA, and CGREF1 between colorectal cancer tissue and normal tissue was verified on the GEPIA website. Abbreviations: OS, overall survival; READ, rectal cancer; COAD, colon cancer; EOCRC, early-onset colorectal cancer

High expression of *KLF9* (Fig. 6A) and *INHBA* (Fig. 6B) indicated markedly poorer outcomes, while high expression of *CGREF1* (Fig. 6C) and *MCM2* (Fig. 6D) indicated better outcomes in the EOCRC cohort. We confirmed the expression of these genes between colorectal tumors and nontumor tissues (Figs. 6E-H) on the GEPIA website. GEPIA PCA established that the 4 cell cycle model genes could remarkably distinguish normal tissues from colorectal cancer samples (Supplementary Fig. 1E).

**Fig. 7:**
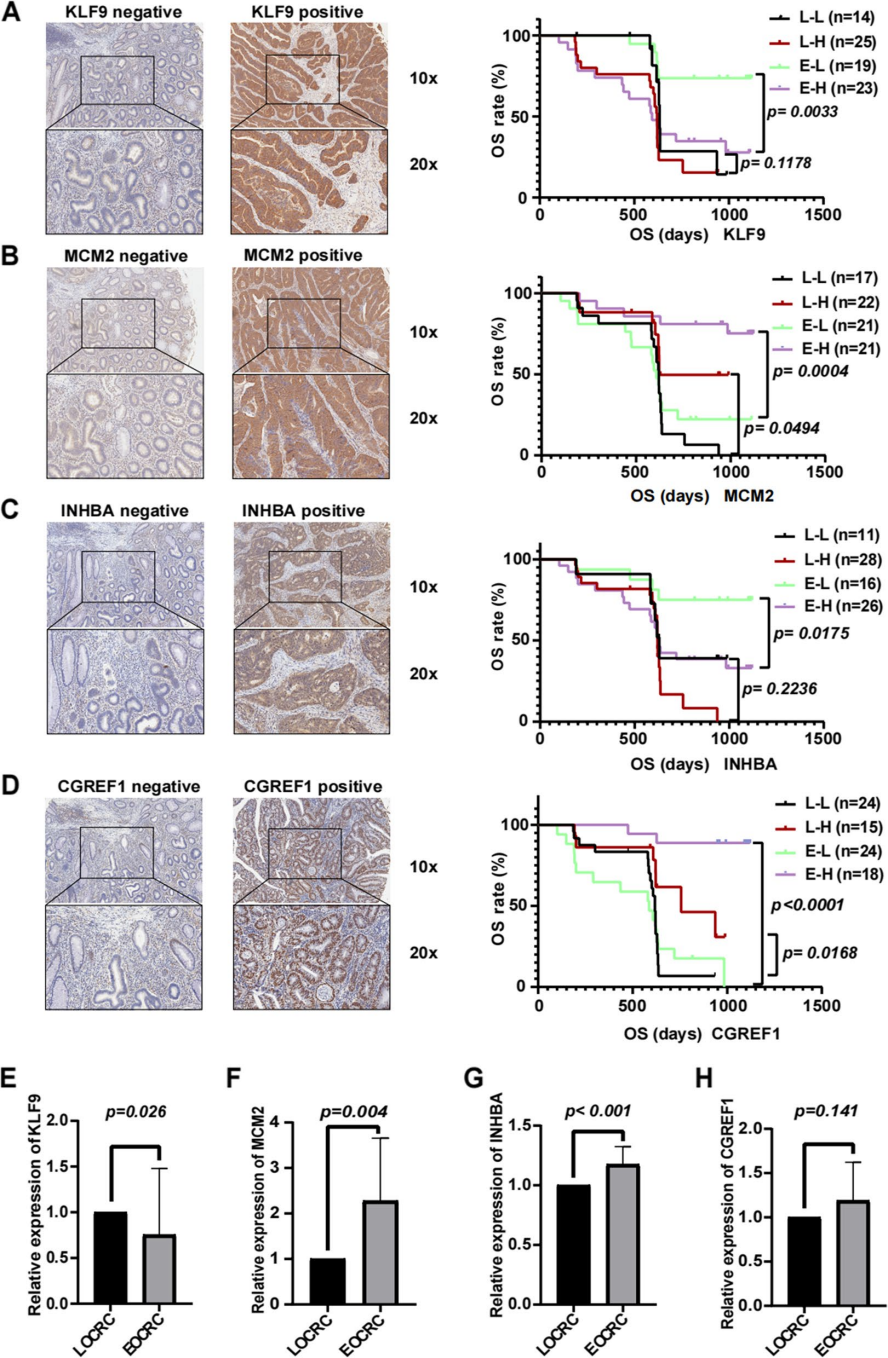
Experimental verification of the expression levels of MCM2, KLF9, INHBA, and CGREF1 and their prognostic significance. **A-D** The difference in the expression levels of MCM2, KLF9, INHBA, and CGREF1. Their prognostic significance was more obvious in the EOCRC cohort than in the LOCRC cohort. **E–H** The expression of MCM2, KLF9, INHBA, and CGREF1 in EOCRC and LOCRC tissues was determined by qPCR. Abbreviations: LOCRC, late-onset colorectal cancer; EOCRC, early-onset colorectal cancer; OS, overall survival; E–H, high-expression early-onset colorectal cancer; E-L, low-expression early-onset colorectal cancer; L–H, high-expression late-onset colorectal cancer; L-L, low-expression late-onset colorectal cancer; IHC, immunohistochemistry analysis; HR, hazard ratio

To verify our findings, a CRC cohort including 42 EOCRC tissues and 39 LOCRC tissues was used for immunohistochemistry (IHC) to explore the clinical significance of the 4 genes. In our study, the expression of KLF9, MCM2, INHBA, and CGREF1 was significantly correlated with TNM clinical stage and tumor differentiation, excluding tumor location, age, and sex (Table 2). Furthermore, we investigated the prognostic value of CGREF1, MCM2, KLF9, and INHBA expression in CRC. We found that CRC patients with higher expression of MCM2 (HR = 0.093, 95% CI = 0.024–0.360, *p* = 0.001) and CGREF1 (HR = 0.104, 95% CI = 0.025–0.438, *p* = 0.002) had significantly better OS than those who had lower expression. However, the expression of KLF9 (HR = 2.788, 95% CI = 0.880–8.831, *p* = 0.081) and INHBA (HR = 1.875, 95% CI = 0.566–6.208, *p* = 0.303) was not related to OS. Interestingly, as shown in Table 2 and Fig. 7A, in the EOCRC cohort, OS differences existed between the high and low KLF9 expression groups (HR = 3.980, 95% CI = 1.683–9.410, *p* = 0.003), although these differences were not present in the LOCRC cohort (HR = 1.939, 95% CI = 0.889–4.230, *p* = 0.118). Similarly, higher expression of INHBA also indicated poor OS in young CRC patients (HR = 3.439, 95% CI = 1.455–8.130, *p* = 0.018) but not in LOCRC patients (HR = 1.719, 95% CI = 0.764–3.869, *p* = 0.224) (Fig. 7C). A more obvious OS difference was observed among different MCM2 and CGREF1 expression groups in the EOCRC cohort than in the LOCRC group [MCM2 in EOCRC (HR = 0.202, 95% CI = 0.084–0.485, *p* < 0.001) and in LOCRC (HR = 0.435, 95% CI = 0.201–0.941, *p* = 0.049) (Fig. 7B); CGREF1 in EOCRC (HR = 0.078, 95% CI = 0.028–0.219, *p* < 0.001) and in LOCRC (HR = 0.380, 95% CI = 0.176–0.819, *p* = 0.017) (Fig. 7D)]. Therefore, we inferred that MCM2, KLF9, INHBA, and CGREF1 may play important roles in the prognosis of EOCRC.

**Table 2:** The correlation between gene expression and the clinicopathologic features of the CRC IHC cohort

		**EOCRC (OS)**		**LOCRC (OS)**		**TNM stage**			**Tumor Differentiation**		
		**HR (95% CI)**	***P* value**	**HR (95% CI)**	**P value**	**I + II**	**III + IV**	***P* value**	**Moderate or Well**	**Poor**	***P* value**
**CGREF1**	**Low expression**	Ref		Ref		19	26		4	44	
	**High expression**	0.078 (0.028–0.219)	< 0.001	0.380 (0.176–0.819)	0.017	23	13	0.023	17	16	< 0.001
**INHBA**	**Low expression**	Ref		Ref		20	7		12	15	
	**High expression**	3.439 (1.455–8.130)	0.018	1.719 (0.764–3.869)	0.224	22	32	0.005	9	45	0.005
**MCM2**	**Low expression**	Ref		Ref		16	27		5	38	
	**High expression**	0.202 (0.084–0.485)	< 0.001	0.435 (0.201–0.941)	0.049	26	12	0.005	16	22	0.002
**KLF9**	**Low expression**	Ref		Ref		24	9		14	19	
	**High expression**	3.980 (1.683–9.410)	0.003	1.939 (0.889–4.230)	0.118	18	30	0.002	7	41	0.005

Abbreviations: *LOCRC* late-onset colorectal cancer, *EOCRC* early-onset colorectal cancer, *CRC* colorectal cancer, *IHC* immunohistochemistry analysis, *HR* hazard ratio, *OS* overall survival, *Ref* reference

Subsequently, we measured the expression of the 4 mRNAs in 6 EOCRC and 6 LOCRC samples by qRT‒PCR. We found that KLF9 (*p* = 0.026) was expressed to a lesser extent in EOCRC, while MCM2 (*p* = 0.004) and INHBA (*p* < 0.001) were more highly expressed in EOCRC than in LOCRC. CGREF1 (*p* = 0.002) expression was not significantly different (Figs. 7E-H). The different expression levels of KLF9, MCM2, INHBA, and CGREF1 seem to be related to age.

## Discussion

Over the past several decades, the morbidity of EOCRC has increased annually and has drawn widespread attention because EOCRC is metastatic, highly malignant, and usually diagnosed at an advanced stage [2]. A growing body of evidence has authenticated the heterogeneity of EOCRC compared with LOCRC [8, 17, 28], and previous prognostic models may not be applicable. In this study, we first collected 273 qualified CRC samples and found that EOCRC seemed to be related to advanced tumor stage and poor survival. Thus, the identification of effective biomarkers for EOCRC prognosis is urgently needed. First, the cell cycle signaling pathway was identified as a significantly enriched oncological signature in EOCRC in our study. Then, we constructed an accurate 4-gene cell cycle classifier. The classifier performed well in both the GEO training cohort and the TCGA validation cohort, which supports the repeatability and utility of the classifier for OS in EOCRC. According to the risk score, patients were divided into high-risk and low-risk groups. We found that the high-risk subgroup had poorer survival than the low-risk group and presented advanced TNM stage, downregulation of DNA damage repair abilities, and an immunosuppressive state. Finally, we verified that the expression levels of KLF9, MCM2, INHBA, and CGREF1 were related to age, tumor stage, and differentiation and were closely related to the prognosis of young CRC patients.

Using GSEA, we found that cell cycle-related gene clusters were upregulated in EOCRC compared with the normal groups. This is consistent with previous research that aimed to determine the molecular characteristics of EOCRC based on proteomics [43]. Cell cycle proteins have been widely reported to be involved in the occurrence and development of tumors [27]. The cell cycle is highly associated with the entire process of cancer. The continuous proliferation of cancer cells is caused by mutations that prevent cell cycle exit, which is the hallmark of cancer [23]. Cell cycle progression is regulated by checkpoint controls and sequential activation of CDKs [44]. Dysregulated cell cycle regulators play an important role in diverse carcinomas and are a hot research direction [27, 44, 45]. In colorectal cancer, previous studies have also demonstrated that the cell cycle plays a vital role in the initiation and progression of cancer. For example, Yi Pend et al. [46] found that the cell cycle G1/S transition was promoted by E26 transformation-specific variant transcription factor 5 (ETV5), which is related to the cell cycle by inhibiting the transcription of p21, thereby accelerating colorectal cancer (CRC) angiogenesis. Xiaoqian Jing et al. [47] explored the activation mechanism of PRPS1 in cell cycle progression to promote tumorigenesis in colorectal carcinoma. For young adults, the cell division rate in healthy human tissues is significantly higher than that in the elderly [48]. Therefore, cancer in elderly individuals also exhibits slower growth than that in young individuals, as their bodies already experience a slower rate of cell development [43, 49]. Yamashita et al. also identified that the WiNTRLINC1/ASCL2/c-Myc axis, which is important for the viability of colon cancer cells, is unique to early-onset differentiated colon cancer [50]. The malignancy of a tumor is directly proportional to the number of proliferative cells in the cancer tissue. This may explain why cancer grows rapidly and is more aggressive in young CRC patients.

The capability of minichromosome maintenance protein 2 (MCM2) to localize to the nucleus in eukaryotic cells is necessary for helicase activity in DNA replication [51]. High expression of *MCM2* was reported to be positively correlated with Ki67 in various malignant tumors, such as CRC, which indicates its crucial carcinogenic role in promoting tumor proliferation [52–54]. Previous studies have indicated that the overexpression of MCM2 promotes CRC cell proliferation and that silencing of MCM2 inhibits cell proliferation by affecting G1/S transition [52]. MCM2 may play a more important carcinogenic role in EOCRC due to the increased proliferation rate and MCM2 expression level in EOCRC vs. LOCRC. Interestingly, MCM proteins are good prognostic markers in many cancers. This may be because of their aberrant expression, a feature of cell cycle disorder that promotes tumorigenesis in cells [55, 56]. A previous study confirmed that high MCM2 expression is associated with a better prognosis in CRC [56]. In this research, the difference in the expression level of MCM2 and prognosis was more significant in the EOCRC cohort than in the LOCRC cohort.

Krüppel-like factor 9 (KLF9) is a member of the SP/KLF family of DNA-binding transcriptional regulators [57], which can regulate various cellular functions such as proliferation, apoptosis, and differentiation. KLF9 is expressed at low levels in CRC [58]. The upregulation of KLF9, a tumor suppressor in pancreatic cancer, may inhibit the progression of this cancer [59]. In previous studies, KLF9 suppressed human breast cancer invasiveness by downregulating matrix metalloproteinase 9 transcription [60] and suppressed the invasion and metastasis of gastric cancer cells by inhibiting the transcription of MMP28 [61]. KLF9 modulates canonical IFN-stimulated genes in the gastrointestinal epithelium through transcriptional inhibition to suppress tumors [62]. Moreover, KLF9 is regulated by CircNOL10, a sponge of miR-135a/b-5p, to suppress the progression of CRC [63]. Notably, as a downstream target of NRF2, KLF9 plays an important role in oxidative stress [64, 65]. Recent research reported that alterations in the NRF2-mediated oxidative stress response may play a distinct role in EOCRC, which emphasizes the potential of modulating oxidative stress as a preventive and therapeutic target for EOCRC [14]. Consistently, our results also suggested that KLF9 plays a significant role in the prognosis of young-onset CRC patients, while this same role was not observed in elderly patients. The mechanism of KLF9 in the oxidative stress response of EOCRC requires further study.

Inhibin βA (INHBA) is a member of the transforming growth factor-β superfamily. This protein has been found to be overexpressed and to promote cell proliferation, invasion, and metastasis in many cancer types [66–68]. High INHBA expression in CRC indicates poor survival [69], which was similar to our findings, especially in EOCRC.

Cell growth regulator with EF-hand domain 1 (CGREF1) is regulated by *p53* and inhibits cell proliferation [70, 71]. Mechanistically, CGREF1 can significantly inhibit the transcriptional activity of AP-1, and its overexpression inhibits the phosphorylation of ERK and p38 MAPK and suppresses the proliferation of HEK293T and HCT116 cells [71]. Nevertheless, the biological function of *CGREF1* has not yet been fully explored, and further research is warranted.

These four genes are all closely related to tumor prognosis, as they regulate tumorigenesis and tumor development through the cell cycle pathway. In our study, the expression of these genes was determined to be related to tumor pathological stage and differentiation and was an independent prognostic factor in early-onset colorectal cancer patients. Notably, we found that KLF9 and INHBA were associated with prognosis in EOCRC but not in LOCRC, while the expression differences in KLF9, MCM2, and INHBA between the tumor and normal groups were more obvious in EOCRC than in LOCRC. The prognostic value of MCM2, KLF9, INHBA, and CGREF1 was closely related to age. Therefore, we inferred that these genes may play more important roles in the prognosis of young CRC patients.

We explored the reasons for the difference in prognosis between the high-risk and low-risk groups identified by our classifier. The results of the GSVA demonstrated that some DNA damage repair pathways that benefit from tumor inhibition, such as mismatch repair, base excision, and homologous recombination, were downregulated in the high-risk group. This might lead to rapid deterioration of the quickly proliferating EOCRC. Moreover, we speculated that defects in DNA damage repair may have a greater impact on the prognostic risk in EOCRC, which exhibits rapid growth with a high rate of cell proliferation. Another important finding of our research was that *PIK3CA* mutations, which were present in 15% of metastatic CRC cases [72, 73], were more common in the high-risk EOCRC samples. A preview study on whole-genome profiling reported that PI3K-AKT pathway genes were upregulated in EOCRC compared with LOCRC [74, 75]. EOCRC patients were more likely to have *PIK3CA* mutations than their older counterparts [76]. Moreover, EOCRC often metastasizes. We speculated that metastasis caused by *PIK3CA* mutations was common in EOCRC patients. *PIK3CA* mutations are usually considered to be closely related to advanced tumor stage and poor survival [77]. Therefore, PI3KCA might be a potential target in metastatic EOCRC. We also found that patients in the high-risk group exhibited immunosuppression, as they had fewer CD8 T cells, activated CD4 T cells, and dendritic cells compared with patients in the low-risk group. Many studies have shown that the density of CD8 + TILs and their antitumor cytotoxic function are related to the long-term survival of patients with different types of cancer [78, 79]. Dendritic cells belong to the innate immune system and are phagocytes that exist in tissues and come into contact with the external environment. They can recognize tumor antigens and present them to cytotoxic T cells, thereby killing cancer cells [80]. Young patients with colorectal cancer, especially those with rectal cancer, have a more pronounced innate immune response, with an increase in complement and acute phase reactants [81]. The low number of dendritic cells in the immune microenvironment of high-risk groups with early-onset colorectal cancer suggests that the population's innate immunity may be impaired and its ability to kill tumor cells may be weakened, thereby affecting prognosis. However, CD4 + TILs play dual roles in tumor progression. In our study, the number of infiltrating CD4 + T cells was lower in the low-risk group. Thus, we supposed that antitumor CD4 + T cells were dominant in EOCRC, which contributes the high proportion of low-risk patients in the EOCRC. However, further experiments are needed. Since immunity decreases with age [82], EOCRC patients may be more sensitive to immunotherapy than their older counterparts [4]. It could be speculated that the low-risk cohort in our study who had a more robust peritumoral immune response would be more likely to benefit from immunotherapy. These findings preliminarily reveal potential reasons for different prognoses between the high- and low-risk groups of EOCRC patients, and these reasons include DNA damage repair, gene mutation, and tumor immunity.

Although the cell cycle-based signature was shown to be an effective independent prognostic factor, some limitations should still be acknowledged. Due to the limited data on early-onset colorectal cancer, our study included only 202 samples. Therefore, our results should be verified by multicenter prospective studies with larger sample sizes. In addition, further studies in vitro and in vivo, such as those involving patient-derived organoids and patient-derived tumor xenografts, are warranted to verify our 4-gene classifier in the near future.

## Conclusion

Due to the poor prognosis of EOCRC patients, effective predictive indicators are urgently needed. However, the heterogeneity of EOCRC limits the applicability of existing models. Thus, a novel prognostic classifier based on cell cycle profiles in EOCRC was developed and validated in our study. We also found that *MCM2, INHBA, CGREF1,* and *KLF9* play critical roles in EOCRC progression. This signature may be used as an important supplement to achieve individualized tumor treatment by optimizing prognosis evaluation.

## Supplementary Information

**Additional file 1:** **Supplementary Figure 1.** (A)HALLMARK and GO analysis of GSEA indentified cell cycle-associated genesets as the most enriched oncological signature of EOCRC cohort in GSE 41258 comparedwith the normal  cohort. (B,C) Before(B) and after(C) batchprocessing of GEO sets. (D) GSVAanalysis indicating downregulation of 9 pathway in high-risk group compare withlow-risk group based on GO set. (E)Normal and colorectal cancer samples could be distinguish definitely by PCAanalysis accroding to the 4 cell cycle- related hub genes. Abbreviations: NES, normalized enrichment score; FDR, false discovery rate; PCA, principal component analysis; READ, rectal cancer;COAD, colon cancer; GSEA, Gene set variation analysisand gene set enrichment analysis; GSVA, Gene Set VariationAnalysis; EOCRC, early-onset colorectal cancer. **Supplementary Table 1.** Baselinecharacteristics of early-onset colorectal cancer. patients in the GEO and TCGAcohorts. Abbreviations: GEO, GeneExpression Omnibus database; TCGA, The Cancer Genome Atlas  database. **Supplementary Table 2.** 98 commondifferentially expressed cell-cycle genes of early-onset colorectal cancer. **Supplementary Table 3.** Unicox genes of early-onset colorectal cancer. **Supplementary Table 4.** The sequences  of  qPCR primers.

## Data Availability

Some of the gene expression data provided in this study are downloaded from the data sets GSE41258, GSE39582, GSE17536, GSE17537, and GSE12945 in the Gene Expression Omnibus database, and can be obtained the following URLs:https://www.ncbi.nlm.nih.gov/geo/query/acc.cgi?acc=GSE41258,https://www.ncbi.nlm.nih.gov/geo/query/acc.cgi?acc=GSE39582,https://www.ncbi.nlm.nih.gov/geo/query/acc.cgi?acc=GSE17536,https://www.ncbi.nlm.nih.gov/geo/query/acc.cgi?acc=GSE17537,https://www.ncbi.nlm.nih.gov/geo/query/acc.cgi?acc=GSE12945. The other gene expression data provided in this study can be downloaded from the cancer genome atlas database at the following website: https://portal.gdc.cancer.gov/.

## Abbreviations

AUC: Area under the curve
CI: Confidence interval
CRC: Colorectal cancer
COAD: Colon cancer
DEGs: Differetial expression genes
EOCRC: Early-onset colorectal cancer
FDR: False discovery rate
GEO: Gene Expression Omnibus database
GSEA: Gene set enrichment analysis
GSVA: Gene set variation analysis
HR: Hazard ratio
IHC: Immunohistochemistry analysis
LASSO: Least absolute shrinkage and selection operator
LOCRC: Late-onset colorectal cancer
NES: Normalized enrichment score
OS: Overall survival
READ: Rectal cancer
ROC: Receiver operating characteristic
TCGA: The Cancer Genome Atlas database
TIL: Tumorinfiltrating lymphocyte
