# Supplementary material for: A novel nomogram based on cell cycle-related genes for predicting overall survival in early-onset colorectal cancer
Source: BMC Cancer. 2023 Jun 27;23:595. doi: 10.1186/s12885-023-11075-y (PMC10303343; doi:10.1186/s12885-023-11075-y)
Supplement: Supplementary file 1 — Additional file 1: Supplementary Figure 1. (A)HALLMARK and GO analysis of GSEA indentified cell cycle-associated genesets as the most enriched oncological signature of EOCRC cohort in GSE 41258 comparedwith the normal cohort. (B,C) Before(B) and after(C) batchprocessing of GEO sets. (D) GSVAanalysis indicating downregulation of 9 pathway in high-risk group compare withlow-risk group based on GO set. (E)Normal and colorectal cancer samples could be distinguish definitely by PCAanalysis accroding to the 4 cell cycle- related hub genes. Abbreviations: NES, normalized enrichment score; FDR, false discovery rate; PCA, principal component analysis; READ, rectal cancer;COAD, colon cancer; GSEA, Gene set variation analysisand gene set enrichment analysis; GSVA, Gene Set VariationAnalysis; EOCRC, early-onset colorectal cancer. Supplementary Table 1. Baselinecharacteristics of early-onset colorectal cancer. patients in the GEO and TCGAcohorts. Abbreviations: GEO, GeneExpression Omnibus database; TCGA, The Cancer Genome Atlas database. Supplementary Table 2. 98 commondifferentially expressed cell-cycle genes of early-onset colorectal cancer. Supplementary Table 3. Unicox genes of early-onset colorectal cancer. Supplementary Table 4. The sequences of qPCR primers. [file 12885_2023_11075_MOESM1_ESM.docx]

Supplementary Material

## Supplementary Figures

**Supplementary Figure 1.**


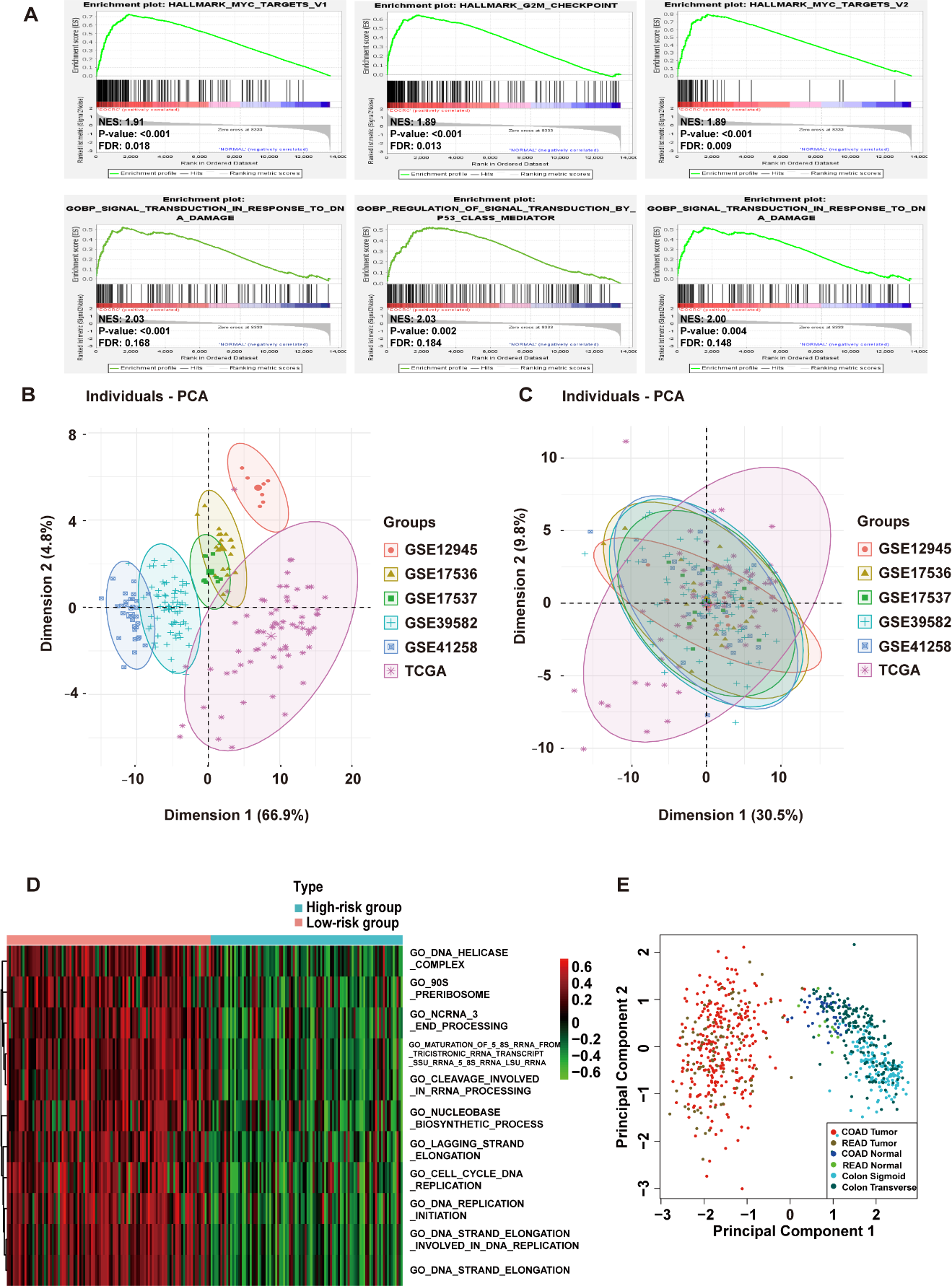


**(A)** HALLMARK and GO analysis of GSEA indentified cell cycle-associated gene sets as the most enriched oncological signature of EOCRC cohort in GSE 41258 compared with the normal cohort. (**B,C)** Before(B) and after(C) batch processing of GEO sets. (**D)** GSVA analysis indicating downregulation of 9 pathway in high-risk group compare with low-risk group based on GO set. **(E)** Normal and colorectal cancer samples could be distinguish definitely by PCA analysis accroding to the 4 cell cycle- related hub genes.

Abbreviations: NES, normalized enrichment score; FDR, false discovery rate; PCA, principal component analysis; READ, rectal cancer; COAD, colon cancer; GSEA, Gene set variation analysis and gene set enrichment analysis; GSVA, Gene Set Variation Analysis; EOCRC, early-onset colorectal cancer.

## Supplementary Tables

**Supplementary Table 1**: Baseline characteristics of early-onset colorectal cancer. patients in the GEO and TCGA cohorts.

| **Characteristics** | **TCGA** | **GSE39582** | **GSE41258** | **GSE17536** | **GSE17537** | **GSE12945** |
| --- | --- | --- | --- | --- | --- | --- |
| **Sample** | N=69 | N=66 | N=31 | N=20 | N=9 | N=7 |
| **Age** |  |  |  |  |  |  |
| **Gender** |  |  |  |  |  |  |
| Female | 39 | 37 | 18 | 6 | 4 | 3 |
| Male | 30 | 29 | 13 | 14 | 5 | 4 |
| **T** |  |  |  |  |  |  |
| T1~2 | 12 |  | 5 |  |  |  |
| T3~4 | 57 |  | 26 |  |  |  |
| **N** |  |  |  |  |  |  |
| N0 | 28 |  | 9 |  |  |  |
| N1 | 25 |  | 9 |  |  |  |
| N2 | 16 |  | 13 |  |  |  |
| **M** |  |  |  |  |  |  |
| M0 | 49 |  | 17 |  |  |  |
| M1 | 20 |  | 14 |  |  |  |
| **Stage** |  |  |  |  |  |  |
| Ⅰ~Ⅱ | 27 | 35 | 7 | 6 | 0 | 4 |
| Ⅲ~Ⅳ | 42 | 31 | 24 | 14 | 9 | 3 |
| **Vital status** |  |  |  |  |  |  |
| Alive | 60 | 49 | 6 | 12 | 5 | 6 |
| Dead | 9 | 17 | 18 | 8 | 4 | 1 |

Abbreviations: GEO, Gene Expression Omnibus database; TCGA, The Cancer Genome Atlas database

**Supplementary Table 2**: 98 common differentially expressed cell-cycle genes of early-onset colorectal cancer.

| ABCB1 | CDT1 | FAP | KLK10 | RRM2 |
| --- | --- | --- | --- | --- |
| ANK3 | CENPE | FGFR2 | MCM10 | SFRP1 |
| AOC3 | CENPF | FOXM1 | MCM2 | SHCBP1 |
| APPL2 | CENPN | GAS2 | MELK | SLC22A3 |
| ARID3A | CEP55 | GDF15 | MET | SLCO1B3 |
| ASNS | CFD | GINS1 | MLXIPL | SMPD3 |
| ASPM | CGREF1 | GINS4 | MSX2 | SOX4 |
| ATAD2 | CKAP2 | GPSM2 | MYC | SOX9 |
| AURKA | CKS2 | HMMR | NEK2 | TIMP1 |
| AURKB | COL7A1 | HPGD | OLR1 | TMEM132A |
| BIRC5 | DACH1 | HSPA2 | PHLDA1 | TOMM34 |
| BMP2 | DLGAP5 | IFIT1 | PLAU | TOP2A |
| BMP4 | DSCC1 | INHBA | PPP1R12B | TPX2 |
| BUB1 | ECT2 | INSM1 | PRKACB | TRIP13 |
| CCNB1 | EDN3 | KIF14 | PTP4A1 | TUBAL3 |
| CCNO | EIF4EBP1 | KIF20A | PTTG1 | UBE2C |
| CDC20 | ENTPD5 | KIF23 | RAD54B | UBE2S |
| CDC25B | ERCC6L | KIF4A | RCAN1 | WNT5A |
| CDK1 | EREG | KLF4 | RECQL4 |  |
| CDKN3 | FANCI | KLF9 | RFC3 |  |

**Supplementary Table 3**：Unicox genes of early-onset colorectal cancer.

| **Characteristics** | **Hazard.Ratio** | **CI95** | **P.value** |
| --- | --- | --- | --- |
| MCM2 | 0.49 | 0.33-0.72 | 2.00E-04 |
| KLF9 | 1.82 | 1.32-2.5 | 3.00E-04 |
| MYC | 0.63 | 0.47-0.84 | 0.0019 |
| AURKB | 0.59 | 0.41-0.84 | 0.0037 |
| INHBA | 1.38 | 1.11-1.71 | 0.0038 |
| OLR1 | 1.27 | 1.08-1.5 | 0.0041 |
| BIRC5 | 0.61 | 0.43-0.86 | 0.0043 |
| UBE2S | 0.6 | 0.42-0.86 | 0.005 |
| CDC20 | 0.65 | 0.48-0.88 | 0.0052 |
| CDT1 | 0.62 | 0.43-0.89 | 0.0094 |
| GINS1 | 0.68 | 0.5-0.93 | 0.0146 |
| CCNB1 | 0.69 | 0.51-0.93 | 0.0147 |
| CGREF1 | 0.69 | 0.51-0.93 | 0.0162 |
| AOC3 | 1.33 | 1.05-1.7 | 0.0194 |
| MCM10 | 0.67 | 0.48-0.94 | 0.0222 |
| RECQL4 | 0.61 | 0.4-0.93 | 0.0222 |
| MELK | 0.67 | 0.47-0.94 | 0.0227 |
| MLXIPL | 0.69 | 0.49-0.97 | 0.0319 |
| TUBAL3 | 0.79 | 0.63-0.99 | 0.0379 |
| EIF4EBP1 | 0.63 | 0.41-0.98 | 0.0386 |
| GINS4 | 0.74 | 0.55-0.99 | 0.0457 |
| FAP | 1.23 | 1-1.5 | 0.0461 |
| FOXM1 | 0.72 | 0.52-1 | 0.0483 |

**Supplementary Table 4**: The sequences of qPCR primers.

| **Gene** | **Primer sequences** | | | |
| --- | --- | --- | --- | --- |
|  | **Forward** | | | **Reverse** |
| **MCM2** | CAGAGCATCTCCATCTCGAAG | | GATGTCAAAGCGTGAGATGATG | |
| **INHBA** | GGCAAGTTGCTGGATTATAGTG | | CTGAGAGTTGGGTACATCCTTT | |
| **CGREF1** | TCCTCTACCTCTTTGCCCTCCATG | | AGCACTTTGTCCACTATCAAGATCACC | |
| **KLF9** | GTGTCTGGTTTCCATTTCGAAC | GATCCCATATCCTCATCTGGAC | | |
| **GAPDH** | TGACTTCAACAGCGACACCCA | CACCCTGTTGCTGTAGCCAAA | | |
